# Something Seems Fishy: Hepatic Abscess Due to Foreign Body Ingestion in a Pediatric Patient

**DOI:** 10.7759/cureus.67205

**Published:** 2024-08-19

**Authors:** James Rhodes, Amanda Macias, Emma Cooke, Joel Jacob, Ashley Devoll, Chelsea Young, Bradford Nguyen

**Affiliations:** 1 Pediatrics, Baylor College of Medicine, Houston, USA; 2 Pediatric Hospital Medicine, Texas Children’s Hospital, Houston, USA

**Keywords:** pediatric case, syndrome of fever of unknown origin, migratory fishbone, hepatic abscess, swallowed foreign body

## Abstract

Pediatric hepatic abscesses are uncommon in children. They are usually preceded by intra-abdominal infections or caused by acute or chronic biliary disease. Cases of hepatic abscesses secondary to foreign body ingestion are even rarer but are most reported in countries such as China, where ingestion of fish and chicken bones is common. We report a rare case of an adolescent patient who developed a hepatic abscess after ingestion of a fishbone foreign body. He presented to the emergency department with emesis, abdominal pain, and subjective fevers of unknown etiology. Initial imaging of the abdomen was pertinent for a heterogeneous hepatic mass with evidence of fluid collection, concerning for malignancy. Subsequent incision and drainage then confirmed fluid collection to be pus. However, his cryptogenic hepatic abscess was not responsive to broad-spectrum intravenous antibiotics. After imaging was re-reviewed and repeated, a 4.3 cm thin curvilinear hyperdensity was identified embedded in the liver parenchyma. Eventually, the patient underwent exploratory laparoscopy where a fishbone foreign body was removed. To our knowledge, this is one of the few reported pediatric cases of hepatic abscess formation caused by a foreign body ingestion. Hepatic abscesses that do not resolve with antibiotics and ultrasound-guided drainage via catheter should prompt reassessment of other uncommon etiologies, specifically migrated foreign bodies as a rare but important differential diagnosis. Compared to pyogenic hepatic abscesses, hepatic abscesses secondary to foreign bodies require expedited surgical intervention for source control; thus, timely recognition and prompt intervention are crucial to minimize morbidity and mortality.

## Introduction

Hepatic abscesses are rare in the pediatric population but are most often reported in individuals with biliary or pancreatic disease, intra-abdominal infections, and those in immunocompromised states such as malignancy [1]. The incidence of pyogenic liver abscesses is 25 per 100,000 pediatric admissions in the United States compared to approximately 80 per 100,000 admissions in developing countries, such as India [2]. Foreign body ingestion is an uncommon cause of hepatic abscesses and further increases diagnostic complexity [3]. In 1898, Lambert was the first to describe a hepatic abscess that formed by ingestion of a foreign body that perforated the gastrointestinal (GI) tract and migrated to the liver [4]. The majority of foreign bodies pass through the GI tract without complications, with 10-20% of foreign bodies requiring non-operative intervention and fewer than 1% requiring surgery [5]. Although several other case reports have identified ingestion of sharp foreign bodies as a cause of treatment-resistant hepatic abscess [3], many cases remain either unrecognized or misdiagnosed as cryptogenic, despite thorough evaluation and imaging [6]. The lack of pediatric case reports and literature describing this phenomenon contributes to this diagnostic gap. Here, we present a case of pediatric hepatic abscess caused by fishbone ingestion with initial misdiagnosis and subsequent treatment failure before eventual surgical removal.

## Case presentation

A 17-year-old male with a four-year history of type II diabetes presented to the emergency department with acute-onset subjective fevers for the past three days and unintentional 15 lbs weight loss over the past three weeks. He also endorsed two weeks of fatigue and one week of epigastric abdominal pain, non-bloody diarrhea, and non-bloody, non-bilious emesis. He denied shortness of breath, chest pain, skin changes, joint pain, and dysuria. He was born in the United States and denied any recent foreign travel; although he was of Nigerian descent. He also denied consumption of raw food, unfiltered water, or contact with animals. Social history was otherwise unremarkable.

On examination, he was febrile to 102.9°F with a heart rate of 108 beats per minute and a respiratory rate of 20 breaths per minute. The abdomen was soft, non-tender, and non-distended. The remainder of the examination was unremarkable. Initial labs were notable for leukocytosis of 14.34 × 10^3^/μL, hemoglobin of 9.5 g/dL (mean corpuscular volume: 74 fL), and platelets of 328 × 10^3^/μL (Table 1). The basic metabolic panel was within normal limits and blood glucose was 122 mg/dL (Table 2). Lipase was elevated to 386 U/L, and liver enzymes were also elevated with aspartate transaminase of 238 U/L, alanine transaminase of 348 U/L, and alkaline phosphatase of 250 U/L (Table 3). He was admitted for further evaluation.

**Table 1:** Cell blood count with platelet and differential.

Laboratory component	Reported results	Reference range and units
White blood cell count	14.34	3.84–9.84 × 10^3^/μL
Hemoglobin	9.5	11.0–14.5 g/dL
Mean corpuscular volume	74.2	76.7–89.2 fL
Hematocrit	29.6	33.9–43.5%
Platelets	328	175–332 × 10^3^/μL

**Table 2:** Basic metabolic panel.

Laboratory component	Reported results	Reference range and units
Sodium	135	136–145 mmol/L
Potassium	4.0	3.5–5.5 mmol/L
Chloride	96	95–105 mmol/L
Carbon dioxide	29	25–35 mmol/L
Calcium	8.9	8.9–10.7 mg/dL
Blood urea nitrogen	8	4–18 mg/dL
Creatinine	0.83	0.60–1.00 mg/dL
Glucose	122	70–100 mg/dL

**Table 3:** Liver panel and lipase.

Laboratory component	Reported results	Reference range and units
Alkaline phosphatase	250	58–237 U/L
Alanine aminotransferase	348	11–26 U/L
Aspartate aminotransferase	238	10–45 U/L
Gamma-glutamyl transpeptidase	334	11–34 U/L
Lipase	386	25–110 U/L

Hospital course

**Figure 1:**
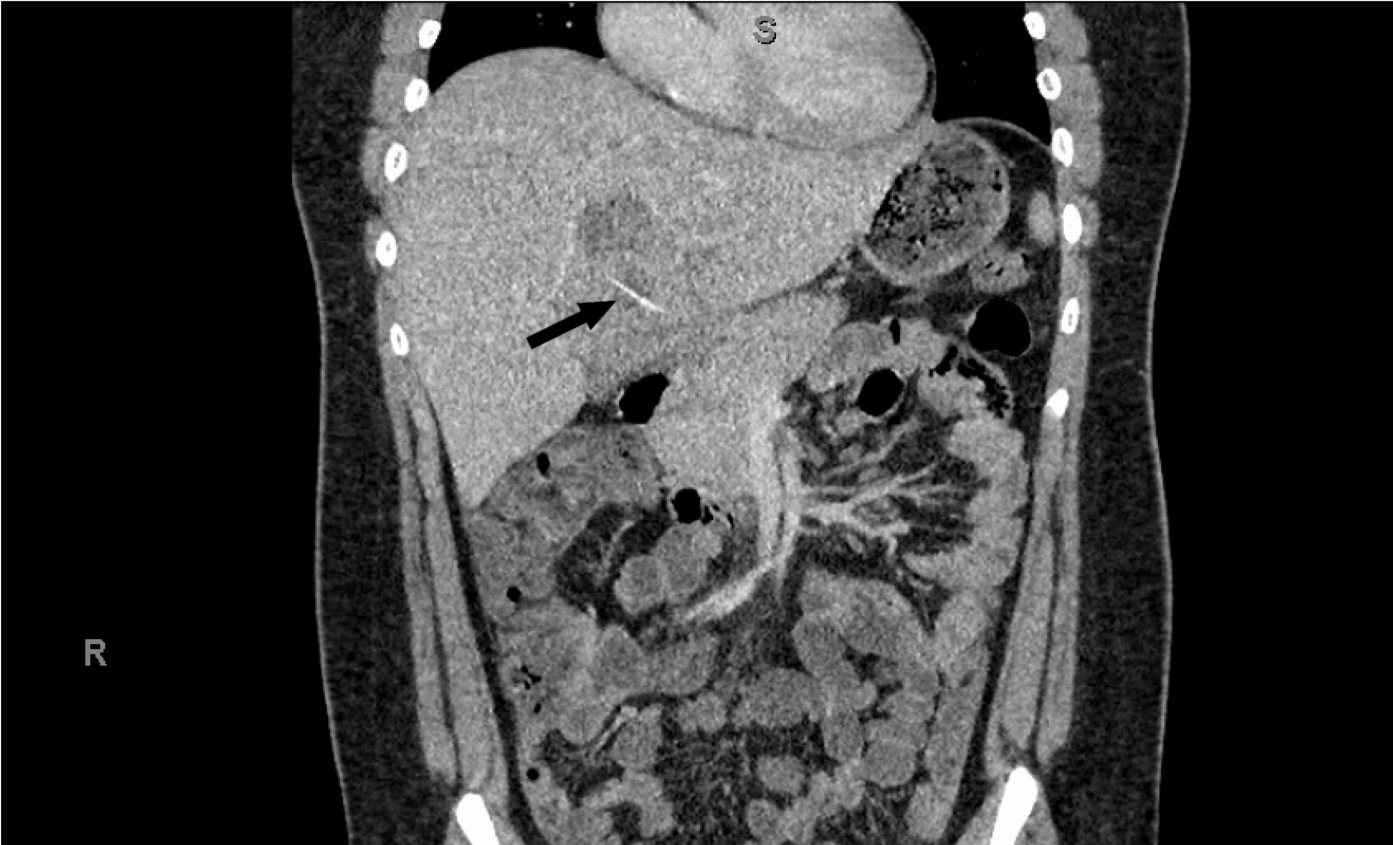
Initial computed tomography (CT) of the abdomen. Initial spiral CT of the abdomen demonstrating an infiltrative heterogeneous hepatic mass thought to be predominately in segment IV. This mass was first characterized to have peripheral calcification, concerning for malignancy. There was no evidence of additional foci of disease within the abdomen. These findings were subsequently amended to include the presence of a thin curvilinear density (black arrow).

Upon admission, the patient had persistent epigastric abdominal pain, so imaging was obtained. Ultrasound (US) of his right upper quadrant (RUQ) was significant for a complex ill-defined lesion in the right hepatic lobe. Computed tomography (CT) of his abdomen demonstrated a heterogeneous hepatic mass initially characterized to have peripheral calcification, concerning for malignancy (Figure 1).

**Figure 2:**
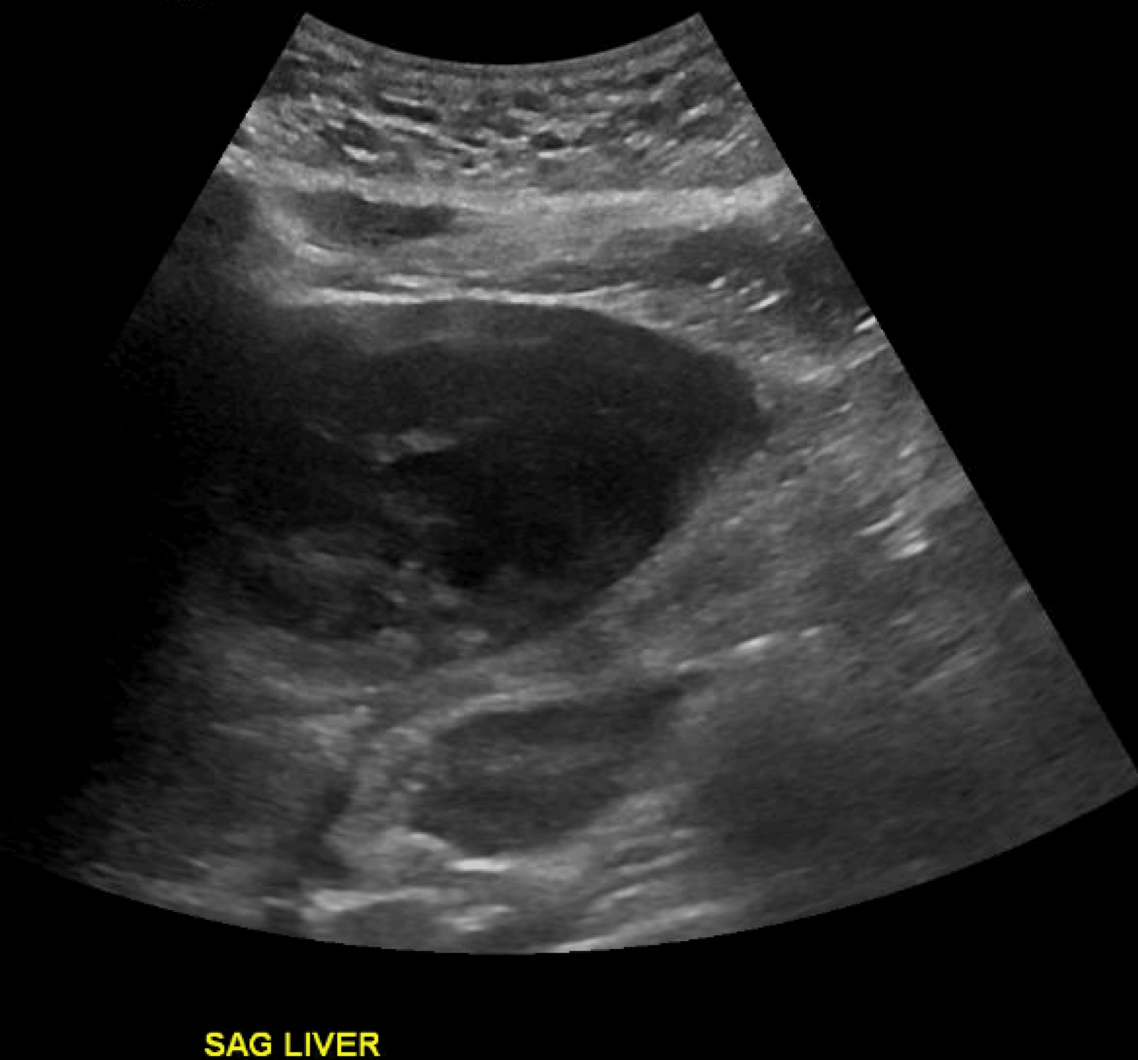
Ultrasound (US) of the right upper quadrant. Ultrasound (US) of the right upper quadrant obtained three days after presentation. Imaging demonstrates an ill-defined, heterogenously large, hypoechoic avascular lesion in the liver with possible fluid or necrosis present in the center. These findings are similar to characteristics appreciated on the initial US obtained on the day of presentation.

The patient was then admitted to the Hematology/Oncology service. Pertinent diagnostic workup included repeat RUQ US demonstrating a large complex hepatic lesion with a possible fluid or necrotic center (Figure 2) as well as normal tumor markers including alpha-fetoprotein and human chorionic gonadotropin. Ceftriaxone was initiated for empiric coverage.

Interventional Radiology was consulted for incision and drainage of the fluid collection. During the procedure, 100 cc of purulent fluid was drained, an exterior drain was placed, and the specimen was sent for culture. Infectious Disease was consulted and antimicrobials were broadened to cefepime, metronidazole, and vancomycin. Within 24 hours of drainage, the patient’s fevers began to improve with cultures subsequently growing *Streptococcus anginosus*, *Streptococcus intermedius*, *Prevotella*, and *Parvimonas micra*. Given that suspicion was now much higher for an infectious etiology, the patient was transferred to the Pediatric Hospital Medicine service for further management.

**Figure 3:**
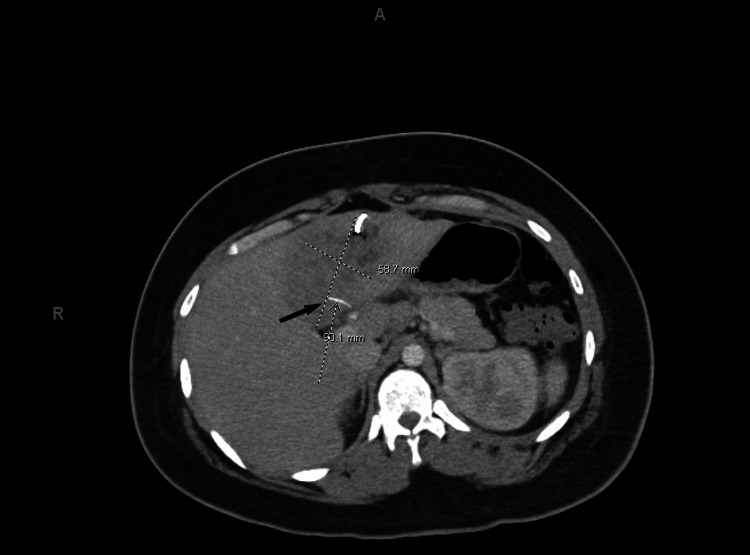
Repeat computed tomography (CT) of the abdomen. Repeat spiral CT abdomen obtained six days after initial imaging. Imaging was obtained after the placement of a pigtail catheter seen in the most anterior aspect of the hepatic collection. The original abscess was still present and only minimally smaller despite drainage. Now, the formation of a small, new abscess was noted in the anterior peritoneum that abuts the rectus abdominis muscle. Lastly, unchanged curvilinear hyperdensity (black arrow) was still present within the collection, concerning for a migrated foreign body as the source of the abscess.

After 10 days of broad-spectrum antibiotics, the patient’s fever curve showed some improvement; however, he did not fully defervesce and continued to have persistent leukocytosis. After struggling to elucidate the underlying etiology in an otherwise previously healthy patient with treatment failure, a closer review of his initial CT scan showed the presence of a thin curvilinear density, and the report was later amended to include this finding (Figure 1). Repeat imaging showed the persistence of the original abscess, the formation of a small, new abscess on the abdominal wall, and the re-demonstration of the curvilinear hyperdensity noted on the first CT scan (Figure 3). By this time, the bacterial polymerase chain reaction (PCR) resulted and detected too many DNA templates that could not be resolved by standard bacterial PCR. After discussions with the Radiology team, the question was raised whether this hyperdensity could be a migrated foreign body acting as a nidus for infection. Moreover, a more focused history later revealed that he regularly consumed fish and recalled eating fish two to three days before fever and presentation.

**Figure 4:**
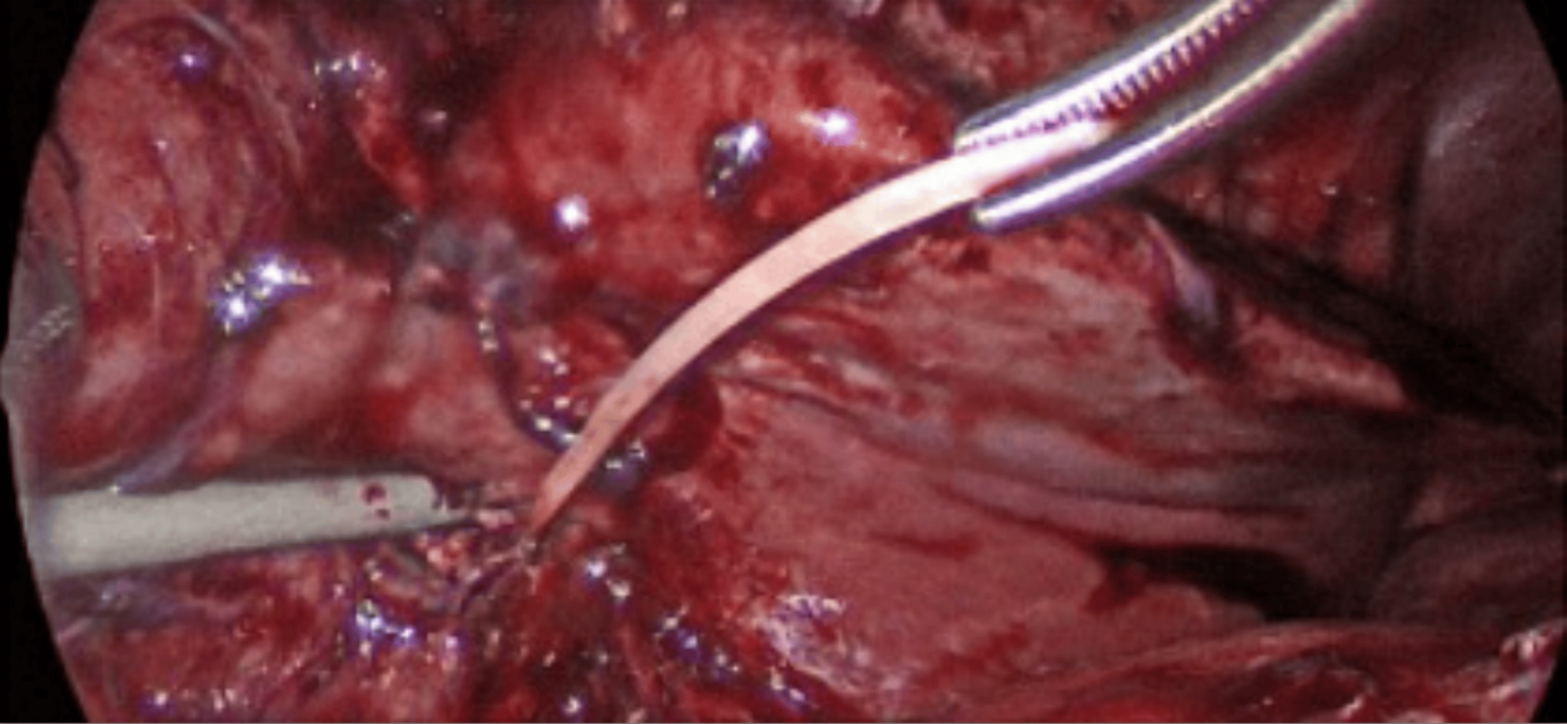
Foreign body during laparoscopy. A foreign body (fishbone) noted on laparoscopy and subsequently removed with partial left liver resection.

**Figure 5:**
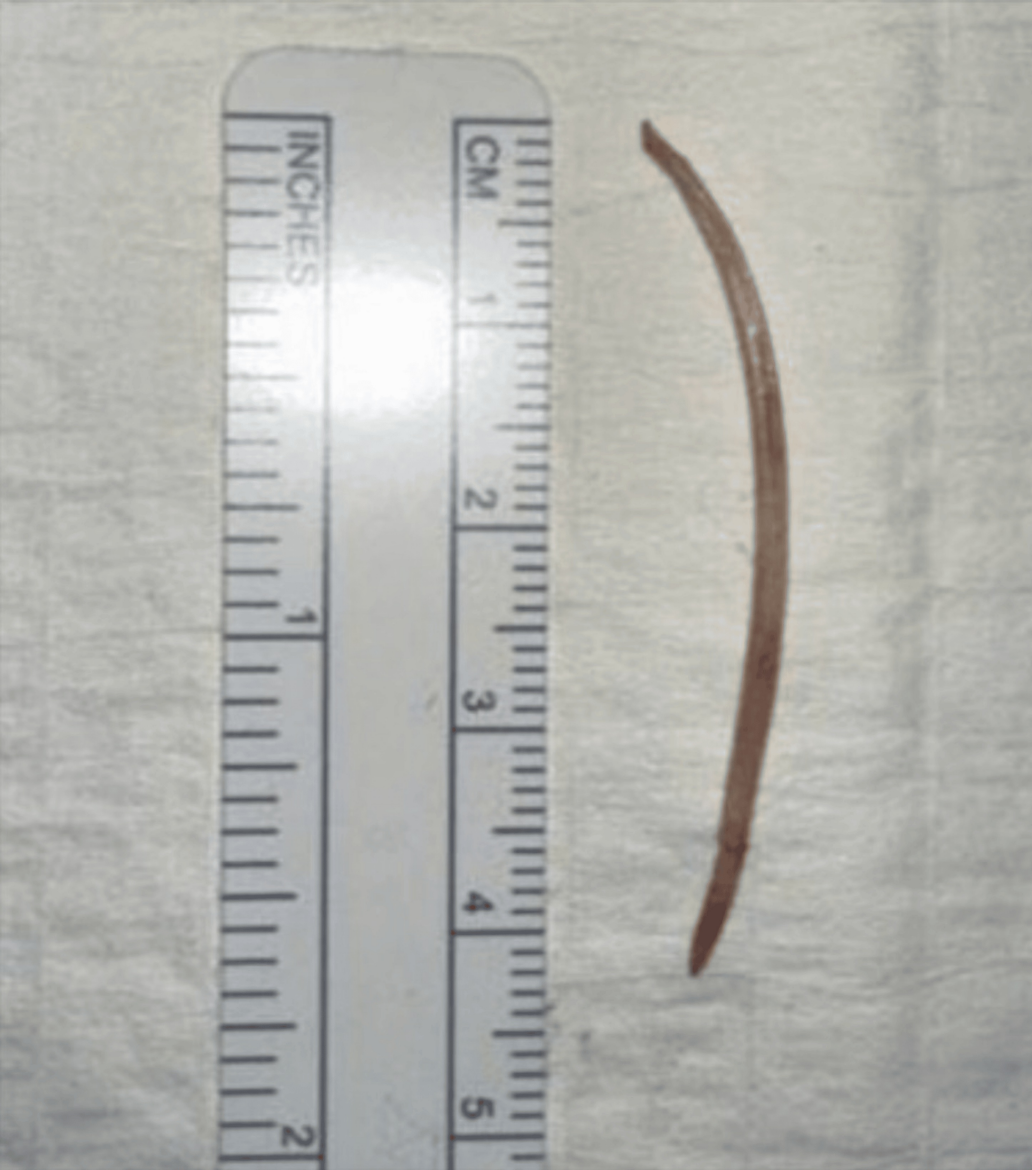
Removed foreign body. The removed foreign body (fishbone) measuring approximately 4.3 cm × 0.1 cm.

Given this hypothesis, Interventional Radiology was reconsulted and a liver biopsy was performed. The biopsy demonstrated chronic inflammation of liver tissue with reactive bile duct proliferation and fibrosis, favoring the diagnosis of an abscess without evidence of malignancy. Although a foreign body was confirmed to be present on imaging, it was unable to be retrieved. Gastroenterology and Pediatric Surgery were then consulted for a combined esophagogastroduodenoscopy with an exploratory laparoscopy for liver wedge resection and foreign body removal. A 4.3 cm curved fishbone foreign body was removed via partial left liver resection (Figures 4, 5). After surgical intervention, the patient’s fevers, leukocytosis, and symptoms fully resolved. He was discharged with an additional two-week course of amoxicillin-clavulanate and Infectious Disease follow-up.

## Discussion

We report a rare presentation of a hepatic abscess caused by a fishbone foreign body. While the majority of ingested foreign bodies will pass through the GI tract without injury, a small number (<1%) can perforate the GI lumen and result in subsequent infection and abscess formation [7]. The stomach is the most frequently documented site of perforation, with other documented sites including the duodenum, ileocecum, and rectosigmoid due to their curvature [3,8]. These sites can be perforated by sharp, rigid objects such as small bones, needles, toothpicks, and pens [3]. Fishbone perforation remains a rare source of hepatic abscesses, though disproportionately reported in foreign countries, such as China, where consumption of fish and chicken bones is more common [9].

Given there are few case reports describing foreign body migration to the liver [3,7-13], delayed diagnosis remains a major concern as presenting symptoms are often vague until abscess formation occurs [3]. Epigastric or vague abdominal pain is the most common symptom on presentation (reported in up to 85% of cases in one systematic review), but other non-specific symptoms can include fever, vomiting, fatigue, and weight loss [3,10]. Abdominal CT or US is the preferred diagnostic modality to identify foreign bodies in the GI tract; however, multiple studies have cautioned against solely relying on imaging for diagnosis due to their limited accuracy [7,11]. In some cases, early endoscopy can be helpful in diagnosis and treatment if performed before foreign body migration and mucosal healing of intraluminal tissue [11,12].

Due to the difficulty of establishing a clear diagnosis of abscess secondary to a foreign body, one multi-center, systematic review proposed a diagnostic algorithm, which aims to enhance a provider’s diagnostic suspicion for foreign body liver abscesses and provide suggestions on the next steps [6]. Once a liver abscess is identified, typical medical management includes initiation of broad-spectrum antibiotics followed by percutaneous drainage. Compared to cryptogenic liver abscesses, migrated foreign body liver abscesses commonly share five of the following characteristics: perforation symptoms (e.g., epigastric pain, upper GI bleeding), absence of underlying conditions (e.g., cancer, immunosuppression), left lobe location, unique location (i.e., only one abscess location within the liver), and adhesions noted during surgery. The same review emphasized that prompt reassessment and consideration of hepatic lobectomy are indicated if patients have less than or equal to two of these characteristics or other red flag indicators, such as evidence of possible foreign body migration on CT and/or treatment failure [6]. Reassessment should include specific history taking regarding foreign body ingestion as well as a meticulous review of prior imaging.

Our patient had four of these characteristics (epigastric pain, absence of an underlying condition, left lobe location confirmed after laparoscopic resection, and a singular location within the liver). Furthermore, we discovered evidence of a curvilinear hyperdensity upon closer review of the initial CT scan, history concerning for foreign body ingestion (recent fish consumption), and treatment failure noted by persistent fevers despite antibiotics and drainage. Additionally, polymicrobial bacterial cultures without known immunosuppression along with a bacterial PCR detecting innumerable DNA fragments should also raise concern for a foreign body. Overall, it is important to safely remove the foreign body promptly after being identified, as delayed diagnosis without proper management significantly increases morbidity and mortality, with many foreign bodies not identified until autopsy [13].

## Conclusions

Diagnosis of a hepatic abscess due to foreign body ingestion is difficult due to non-specific symptoms and the possibility of ambiguous imaging findings. Broad differential diagnoses (such as malignancy, as in our case) may also divert attention and further delay appropriate treatment. After initial diagnostic uncertainty, a multidisciplinary approach led to the correct identification and management of our patient’s hepatic abscess resulting from foreign body perforation of the GI tract. This case highlights the value of multidisciplinary involvement, particularly in cases where diagnosis may be challenging to establish. It also points to the benefit of pursuing further evaluation in cases of cryptogenic infections, particularly in immunocompetent hosts. Despite maximizing non-invasive treatment modalities, ultimately, a surgical approach was required for our patient and provided both definitive diagnostic and therapeutic management. Finally, this case underscores the importance of being culturally sensitive to dietary differences when interviewing patients. Upon taking a more specific history, our patient endorsed both recent and frequent fish consumption, which is reportedly common in Nigerian cuisine.

Literature regarding migrated foreign body liver abscesses in pediatric patients is extremely limited. Unfortunately, most of the literature referenced in this case report pertains specifically to adult populations. As witnessed in our 17-year-old patient, children of all ages may ingest a foreign body, but young children (ages six months to three years) are at the highest risk as they put objects in their mouths to explore the world. Hopefully, this case report will improve the diagnostic gap of migratory foreign body liver abscesses by not only increasing awareness but also promoting further research in pediatric patients of all ages.
